# Supplementary material for: The Effects of Genetic Relatedness on the Preterm Infant Gut Microbiota
Source: Microorganisms. 2021 Jan 29;9(2):278. doi: 10.3390/microorganisms9020278 (PMC7911719; doi:10.3390/microorganisms9020278)

Frequency distribution and correlation matrix of transformed clinical and alpha diversity variables

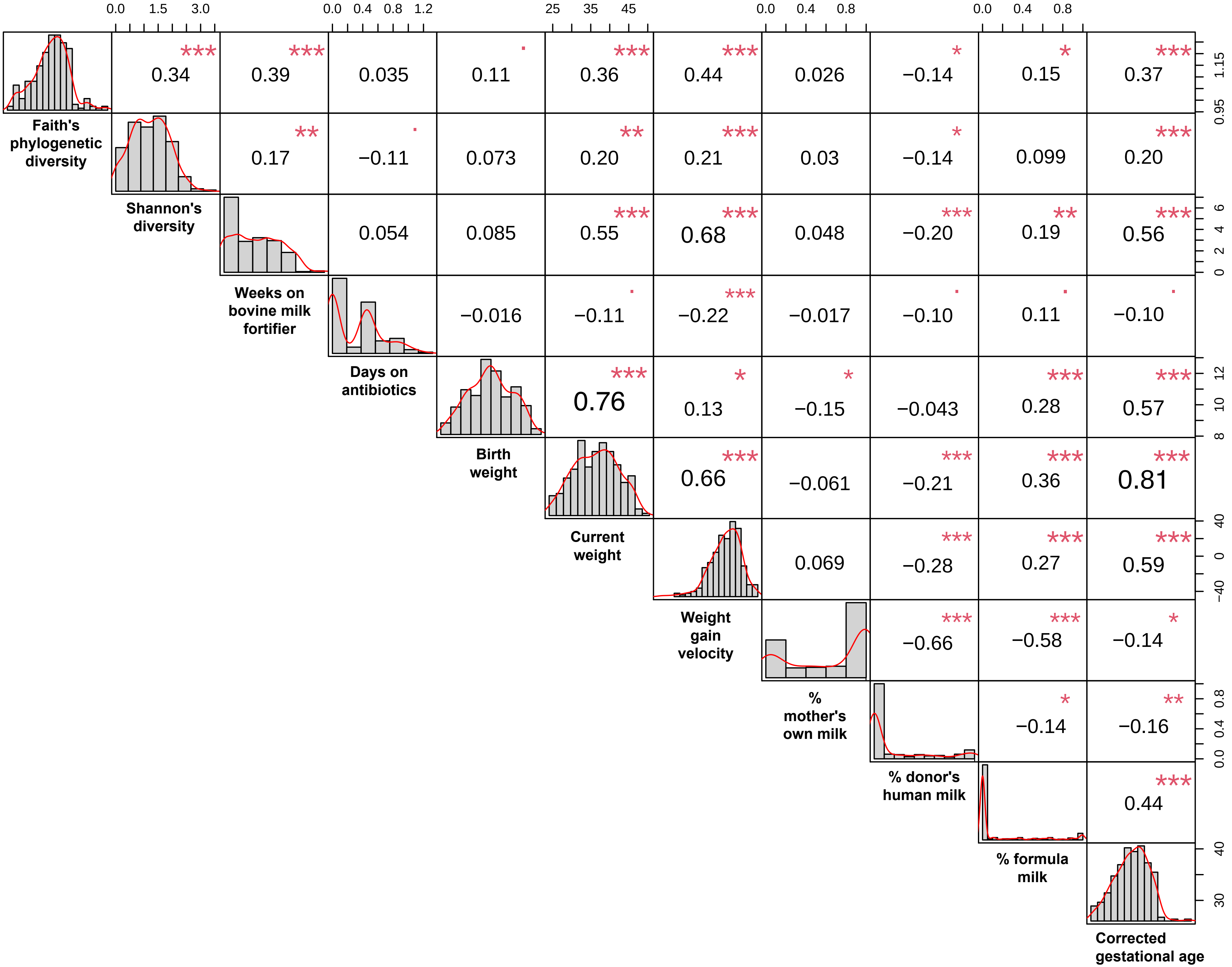

Supplement: Supplementary file 1 [file microorganisms-09-00278-s001.zip › Fig_S1_alpha_transformed_vardist_half.pdf]
