# Supplementary material for: The Effects of Genetic Relatedness on the Preterm Infant Gut Microbiota
Source: Microorganisms. 2021 Jan 29;9(2):278. doi: 10.3390/microorganisms9020278 (PMC7911719; doi:10.3390/microorganisms9020278)
Supplement: Supplementary file 1 [file microorganisms-09-00278-s001.zip › TableS1_SMD.docx]

**Table S1.** List of primers used in this study.

| **Cohort** | **Primer Target**  **(16S rRNA gene)** | **Primer** | **Locus-specific primer sequence**  **(5’->3’)** |
| --- | --- | --- | --- |
| Carle Foundation Hospital | V3-V4 region | V3_F357_N  V4_R805 | CCTACGGGNGGCWGCAG  GACTACHVGGGTATCTAATCC |
|  |  |  |  |
| Tampa General Hospital | V4 region | 515F Modified  806R Modified | GTGYCAGCMGCCGCGGTAA  GGACTACNVGGGTWTCTAAT |
