# Supplementary figures and images for: The Effects of Genetic Relatedness on the Preterm Infant Gut Microbiota
Source: Microorganisms. 2021 Jan 29;9(2):278. doi: 10.3390/microorganisms9020278 (PMC7911719; doi:10.3390/microorganisms9020278)

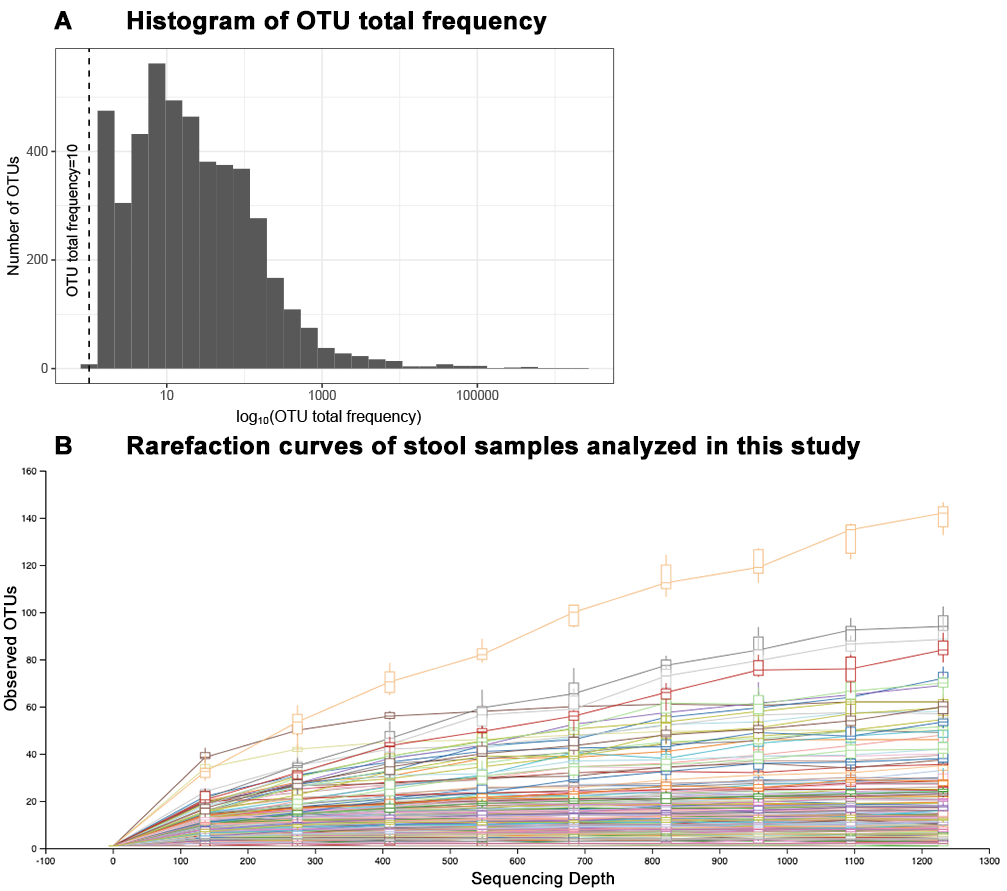

Supplement: Supplementary file 1 [file microorganisms-09-00278-s001.zip › Fig_S2.tif]
